# Detecting Deception and Ensuring Data Integrity in a Nationwide mHealth Randomized Controlled Trial: Factorial Design Survey Study

**DOI:** 10.2196/66384

**Published:** 2025-01-28

**Authors:** Krista M Kezbers, Michael C Robertson, Emily T Hébert, Audrey Montgomery, Michael S Businelle

**Affiliations:** 1 Tobacco Settlement Endowment Trust Health Promotion Research Center Stephenson Cancer Center University of Oklahoma Health Sciences Oklahoma City, OK United States; 2 Department of Family and Preventive Medicine University of Oklahoma Health Sciences Oklahoma, OK United States

**Keywords:** ecological momentary assessment, enrollment, fraud, mHealth, randomized controlled trial, recruitment, deception, data integrity, behavior, social, RCT, factorial design, mobile phone

## Abstract

**Background:**

Social behavioral research studies have increasingly shifted to remote recruitment and enrollment procedures. This shifting landscape necessitates evolving best practices to help mitigate the negative impacts of deceptive attempts (eg, fake profiles and bots) at enrolling in behavioral research.

**Objective:**

This study aimed to develop and implement robust deception detection procedures during the enrollment period of a remotely conducted randomized controlled trial.

**Methods:**

A 32-group (2×2×2×2×2) factorial design study was conducted from November 2021 to September 2022 to identify mobile health (mHealth) survey design features associated with the highest completion rates of smartphone-based ecological momentary assessments (n=485). Participants were required to be at least 18 years old, live in the United States, and own an Android smartphone that was compatible with the Insight app that was used in the study. Recruitment was conducted remotely through Facebook advertisements, a 5-minute REDCap (Research Electronic Data Capture) prescreener, and a screening and enrollment phone call. The research team created and implemented a 12-step checklist (eg, address verification and texting a copy of picture identification) to identify and prevent potentially deceptive attempts to enroll in the study. Descriptive statistics were calculated to understand the prevalence of various types of deceptive attempts at study enrollment.

**Results:**

Facebook advertisements resulted in 5236 initiations of the REDCap prescreener. A digital deception detection procedure was implemented for those who were deemed pre-eligible (n=1928). This procedure resulted in 26% (501/1928) of prescreeners being flagged as potentially deceptive. Completing multiple prescreeners (301/501, 60.1%) and providing invalid addresses (156/501, 31.1%) were the most common reasons prescreeners were flagged. An additional 1% (18/1928) of prescreeners were flagged as potentially deceptive during the subsequent study screening and enrollment phone call. Reasons for exclusion at the screening and enrollment phone call level included having an invalid phone type (6/18, 33.3%), completing multiple prescreeners (6/18, 33.3%), and providing an invalid address (5/18, 27.7%). This resulted in 1409 individuals being eligible after all deception checks were completed. Postenrollment social security number checks revealed that 3 (0.6%) fully enrolled participants out of 485 provided erroneous social security numbers during the screening process.

**Conclusions:**

Implementation of a deception detection procedure in a remotely conducted randomized controlled trial resulted in a substantial proportion of cases being flagged as potentially engaging in deceptive attempts at study enrollment. The results of the deception detection procedures in this study confirmed the need for vigilance in conducting remote behavioral research in order to maintain data integrity. Implementing systematic deception detection procedures may support study administration, data quality, and participant safety in remotely conducted behavioral research.

**Trial Registration:**

ClinicalTrials.gov NCT05194228; https://clinicaltrials.gov/study/NCT05194228

## Introduction

A paradigm shift in methodologies used to conduct randomized controlled trials (RCT) focused on human behavior and behavior change is underway [1]. Specifically, remotely conducted behavioral trials, trials that do not require in-person visits, are increasingly commonplace. This was true before the onset of the COVID-19 pandemic, and this trend has accelerated in recent years due to technological advancements and shifting social norms [2].

By reducing barriers to participation in RCTs (eg, transportation limitations and inability to take off work), remotely conducted trials may have greater generalizability than traditional in-person RCTs and may extend health promotion programs to those who might otherwise lack access to them. Furthermore, the widespread use of smartphones, now owned by 90% of adults in the United States, presents an unprecedented opportunity to provide personally tailored health-promoting content to vast numbers of people [1,3].

There are potential disadvantages to conducting behavioral trials remotely via smartphones. First, obtaining appropriately representative samples to study digitally mediated behavior interventions can be challenging [4,5]. While this issue is not unique to remotely delivered interventions, participation requires a willingness and ability to operate various technologies that may systematically affect inclusion, potentially leaving remotely conducted research prone to limitations imposed by the “digital divide” [6]. For example, lower ownership of smartphones among older adults, Americans with lower incomes, and people who reside in rural environments can limit equitable inclusion of these groups if these realities are not carefully addressed [7].

Second, remotely conducted behavioral trials may be prone to participant deception [8]. Several types of deception among research participants in clinical trials have been identified [8]. Concealment has been defined as the act of failing to disclose relevant information to facilitate study admittance (eg, a potential participant not sharing that they have an exclusionary comorbidity) [8]. *Fabrication* refers to the duplicitous invention on the part of a potential participant to support their inclusion in a research trial (eg, erroneously claiming to be diagnosed with a health-related condition) [8]. *Collusion* is a coordinated sharing of information among potential research participants to gain study admittance [8]. Deception on the part of research participants can lead to considerable waste of study resources, compromised integrity of data resulting from a study, and threats to participant safety [8]. Indeed, severe symptoms and death have been attributed, in part, to deception on the part of research participants [9,10].

Financial gain appears to be a primary reason people engage in deceptive practices to enroll in research studies [11,12]. Participants are commonly paid to participate in research studies to reimburse for out-of-pocket expenses associated with study participation (eg, smartphone data usage), to compensate for time and effort associated with study participation, and to incentivize participation [13]. The prevailing consensus is that paying individuals to participate in research is ethical if it does not undermine informed consent [13]. Qualitative research studies have highlighted the critical role that monetary payment plays in increasing willingness to participate in RCTs [14,15]. However, higher levels of compensation can increase the likelihood of deceptive practices to gain admittance into research studies [15].

Financial incentives may motivate people to serially engage in clinical trials. Devine et al [11] recruited “experienced research participants” (ie, individuals who had participated in more than one study in the past year). Research participants (N=100) reported participating in an average of 12 studies within the previous year (range 2-100 studies), with reported lifetime earnings from research participation to be US $9809 on average (range US $50-US $175,000). A total of 75% of participants in this study reported engaging in concealment to avoid study exclusion, and 25% reported engaging in fabrication to facilitate study inclusion. Qualitative data from this study also characterized instances of collusion, including a description of how “research kingpins” have been known to trade information with individuals to facilitate their admittance into research studies [11]. The researchers suggested that “professional subjects” may be substantially overrepresented in clinical research [11].

Technologies that can greatly extend the reach of remotely conducted behavioral research may leave this type of research especially prone to deception from research participants. The enhanced accessibility of digitally mediated behavioral interventions may disproportionately attract individuals who aim to “game the system” [7]. For example, digital screening processes may be exploited by individuals who repeatedly restart the screening process to change their information to satisfy eligibility requirements [7]. There are reports of individuals accessing ClinicalTrials.gov and other clinical trial repositories to identify study inclusion and exclusion criteria to support deceptive enrollment into RCTs [11]. Remotely conducted behavioral research trials may be especially attractive to experienced study participants (1) because they are often low risk—this appears to be an especially salient consideration among people who participate in many research studies annually [11], and (2) because considerable remuneration is often used as a strategy to reduce the high rates of attrition that can afflict digitally mediated studies [16].

Although it may be impossible to entirely eliminate this threat, there is a growing catalog of techniques for preventing and minimizing the ill effects of deception from research participants in digitally mediated behavioral interventions. Devine et al [11] suggested that limiting the face validity of items used to screen out participants and concealing specific reasons for nonadmittance may reduce deception. Dahne et al [7] created a visual database to aid members of their research team in the identification of potential participants who may have entered numerous screening records. Abroms et al [17] and Bricker et al [18] used techniques such as a CAPTCHA (Completely Automated Public Turing test to tell Computers and Humans Apart) to validate potential participants as human (ie, not automated programs [bots]), to evaluate screening IP addresses (eg, removing all duplicated IP addresses or those not of US origin), and to review system usage data (ie, taking less than 90 seconds to complete a screening survey or taking less 10 minutes to complete the baseline survey). For flagged cases, members of these research teams contacted individuals to further evaluate the authenticity of attempts at study enrollment. Resnik and McCann [19] have recommended that any evidence of potentially deceptive activity should result in the exclusion of the prospective participant from the study enrollment.

Verifying information provided by participants via photo identification, objective measures, and social security numbers (SSNs) may reduce deceptive attempts at study enrollment [8,11,19]. Smartphone-mediated screening processes may facilitate submitting photographs of identification cards. Smoking status can be tested biochemically using devices that can be remotely delivered [8,11,19]. Advising participants that their self-report responses will subsequently be verified may dissuade deception. Incentivizing the provision of accurate information may further support data integrity (eg, providing a “bonus” for providing information that is not discrepant with subsequent objective measures) [19].

This study details techniques that we implemented to minimize the effects of deception from individuals who attempted to enroll in the Exemplar study, a remotely delivered, nationwide RCT. We conclude by providing recommendations for future remotely conducted behavioral interventions.

## Methods

### Study Overview

The Exemplar study used a 2×2×2×2×2 factorial design to determine smartphone survey factors related to high daily survey completion rates. Eligible participants were randomly assigned and completed a baseline survey, 28 days of brief daily ecological momentary assessments (EMAs), and a final survey. The entire study was conducted remotely via REDCap (Research Electronic Data Capture; Vanderbilt University) and the Insight platform [20]. The Insight platform allows researchers to rapidly create, develop, and implement EMA studies [21]. This study was registered with ClinicalTrials.gov (NCT05194228). Details concerning the study design, participants, and outcomes are presented elsewhere [20].

### Recruitment Strategy

The target population for this study was adults (ie, 18 years or older) living in the United States who had an active Android smartphone. Participants were recruited via Facebook advertisements from November 2021 to September 2022. The advertisements were targeted to individuals aged at least 18 years old who possessed an Android smartphone. Examples of Facebook advertisements included:

We’re looking for Android users to complete brief daily surveys on their smartphones. Qualified participants will be compensated up to $152 over 4 weeks. NO in person visits required.” and “Have an Android smartphone? See if you’re eligible to participate in a 4-week research study. Qualified participants will be compensated up to $152 for their time. NO in person visits required.

Clicking on a Facebook advertisement initiated an encrypted REDCap prescreener survey that was used to determine initial eligibility for the study. Individuals were not shown their eligibility results until after all prescreener items were complete. Ineligible individuals were informed that they were not eligible for the study, but the specific reason(s) were not provided. If a participant was potentially eligible based on the REDCap prescreener, they selected times for a screening and enrollment call. Study staff texted eligible participants, thanking them for completing the prescreener and confirming the date and time for their screening and enrollment call.

### Enrollment and Participant Flow

During the screening and enrollment call, individuals were asked additional screening questions; verified that their smartphone was compatible with the Insight mobile health (mHealth) platform [21]; completed a reading comprehension test, Rapid Estimate of Adult Literacy in Medicine – Short Form [22]; and completed the informed consent process. Individuals were also required to text a picture of their photo ID with a US address. Once fully enrolled, participants downloaded the Insight smartphone app onto their personal Android smartphones. They received instructions on how to use the smartphone app to complete the baseline survey, daily EMAs for 28 days, and the final survey. After the baseline survey was completed, participants were mailed a reloadable Greenphire Mastercard. Baseline compensation (ie, US $10) was loaded onto the Greenphire card after the participant confirmed that they received the card by texting study staff the last 4 digits of their card. The rationale for requiring participants to text the last 4 digits of their card was to confirm when they received their card. Funds were loaded onto participant cards after they verified receipt of the card to ensure payment was received (eg, to overcome the possibility of cards being lost in the mail). Compensation for completing EMAs (ie, up to US $56 for those randomly assigned to complete two daily EMAs, up to US $112 for those randomly assigned to complete four daily EMAs) and the final survey (US $30) was loaded onto the Greenphire card at the completion of the study.

### Measures

#### REDCap Prescreener

All individuals who clicked a Facebook advertisement were taken to an encrypted REDCap screening survey hosted by the University of Oklahoma Health Sciences Center (OUHSC). Individuals completed demographic questions (eg, age, race, address, and phone number) and questions about their smartphones (eg, phone ownership and type of phone). Individuals who met the study inclusion criteria were asked to select a primary and secondary time for the screening and enrollment phone call. Deception detection steps, along with associated considerations and decisions, for the REDCap prescreener can be seen in Table 1. These steps address the deception detection review that occurs once an individual is determined to be eligible at the REDCap prescreener level. REDCap tracking allowed for easy reporting and periodic checks of the data to determine if there were any prescreener patterns that could indicate new types of attempts at deceptive enrollment. This was initially how it was determined to add reCAPTCHA and the identity statement (Table 1, step 6).

**Table 1:** Steps taken to detect potential deception in REDCap^a^ prescreeners that were completed by individuals that viewed study advertisements.

Deception detection step for the REDCap prescreener	Considerations and decisions
1. Incomplete prescreeners	Incomplete prescreeners were not reviewed.
2. Duplicative prescreeners	Previous prescreeners with identical information, individuals currently or previously enrolled in the study, and previously ineligible individuals were not scheduled for a screening and enrollment call.
3. Home and mailing address	Validated by Google and Google Maps; invalid addresses and business addresses not linked to an individual’s residence were not scheduled for a screening and enrollment call.
4. Phone number	Phone number provider search was conducted to determine use of bandwidth phones (ie, a phone not connected to a cellular provider); participants were scheduled for a screening and enrollment call but not excluded; this step was collected for future consideration of exclusion.
5. Email address	Verified by Google search as an exploratory approach to deception detection; participants were scheduled for a screening and enrollment call but not excluded.
6. reCAPTCHA^b^ and identity statement	8.5 weeks into the study, reCAPTCHA was added to prevent bots from autofilling the prescreener; 9.5 weeks into the study, an identity statement was added, “Due to a large number of spam accounts, we will verify your identity prior to enrolling you in the study.”
7. Periodic data checking	Data were periodically reviewed to determine potential patterns of deception. Cases that passed the deception deterrence checklist but were flagged as suspicious were scheduled for a screening and enrollment phone call.
8. Conservative decision-making	In cases where there was any doubt about whether a prescreener contained deceptive information, the screening and enrollment call was scheduled. This conservative approach allowed for the reduction in deceptive enrollments while ensuring that eligible individuals could participate in the study.

^a^REDCap: Research Electronic Data Capture.

^b^CAPTCHA: Completely Automated Public Turing test to tell Computers and Humans Apart.

#### Screening and Enrollment Phone Call

During this phone call, individuals reviewed and electronically signed the informed consent through the REDCap link, completed a reading comprehension test [22], provided their SSN, and uploaded a copy of their photo identification. All pictures of identification were deleted after identity verification to reduce potential breaches in confidentiality. Eligible individuals provided a minimum 14-hour window of waking hours for each day of the week (EMAs could only be prompted during waking hours). Table 2 shows the deception detection steps for the screening and enrollment phone call.

**Table 2:** Steps taken to detect potential deception during the study enrollment phone calls.

Deception detection step for the screening and enrollment phone call	Considerations and decisions
9. Informed consent	If the signed name differed from the REDCap^a^ prescreener, study staff asked the individual to confirm the name that was signed. If the name did not match, the screening and enrollment call was terminated, and the screener was marked as “deceptive.” Shortened names (eg, Chris instead of Christopher) were not considered to be deceptive. Married versus maiden names were confirmed by uploading a picture of a piece of stamped mail with the correct name and matching mailing address. If a matching piece of stamped mail could not be provided, the screening and enrollment call was terminated, and the individual was not invited to enroll in the study.
10. Photo identification	The name and address on the individual’s photo identification had to match information provided during the REDCap prescreener. If the address did not match, individuals were required to send a picture of a piece of stamped mail that was sent to them at their address. If no other information could be provided to verify their address, the screening and enrollment call was ended, and the individual was not allowed to enroll in the study.
11. Social security number	SSN^b^ was required to pay research participants at our institution and was needed for tax reporting purposes. If an individual declined to provide their SSN, the screening and enrollment call was ended, and the individual was marked as “Opt to not participate” in REDCap
12. Greenphire Mastercards	Physical Greenphire Mastercards, rather than virtual gift cards, were used to compensate participants for completing study surveys. Greenphire cards were only mailed to the verified mailing address for each participant after they completed the baseline survey in the Insight app.

^a^REDCap: Research Electronic Data Capture.

^b^SSN: social security number.

#### Baseline Survey, EMAs, and Final Survey

Participants used the Insight app to complete the baseline, daily EMAs (2-4 per day), and follow-up surveys. These surveys asked about affect, health, and health behaviors.

#### Compensation

Once a participant received their Greenphire card in the mail, they were instructed to contact study staff to confirm the card had been received. Study staff then sent the participant a link to sign that they had received their card. This was an additional step to ensure that participants lived at the address they provided in their prescreener and that they interacted with study staff for an additional time. Individuals who did not confirm their card did not receive any study payments.

### Statistical Methods

Descriptive statistics were used to describe the sample and highlight deception detection outcomes. All analyses were conducted using SPSS (IBM Corp, version 29).

### Ethical Considerations

The University of Oklahoma Health Sciences Institutional Review Board approved this study (IRB#13684). All participants in this study signed an informed consent via REDCap before engaging in any research study activities. The informed consent provided language that the study is optional and participants could withdraw at any time. All study datasets were deidentified before analyses were conducted. Participants could be compensated up to US $152 depending on the group they were randomized into for the study. Since this paper focused on the steps leading up to the start of the study, no further compensation details are provided, but they can be found in the primary outcomes paper [20].

## Results

From November 29, 2021, to September 27, 2022, Facebook advertisements yielded 5236 clicks and openings of the REDCap screener. Of those screeners, 3308 were ineligible (eg, 1158 blank prescreeners), and 1928 REDCap prescreeners indicated initial eligibility for the study. Of the 1928 eligible screeners, 195 (15.3%) out of 1928 were duplicates of previously eligible individuals, and 206 (10.7%) out of 1928 were marked as potentially deceptive (Table 3). All screening outcomes are presented in Table 3. Ultimately, 1409 individuals remained eligible after deception detection steps 1-8 were implemented. The deception detection checklist required, on average, approximately 1-4 minutes to complete for each eligible REDCap prescreener.

On January 27, 2022, reCAPTCHA was added to prevent bots from autocompleting the REDCap prescreener in rapid succession. On February 2, 2022, an identity statement was added in the REDCap prescreener so individuals were aware their identity would be verified before enrollment. A total of 3 (1.5%) inaccurate SSNs were detected out of 202 participants who earned over US $100, the OUHSC minimum reporting requirement for participant compensation. The SSNs of the 283 participants who earned less than US $100 were not checked. During the screening and enrollment call, 65 (3.4%) out of 1928 pre-eligible individuals declined to provide their SSN and opted to not participate in the study.

A total of 1928 nonduplicate, complete REDCap screeners were examined using our deception detection and abatement procedure. The majority of deceptive attempts at enrolling into the study were detected at the REDCap screener level (501/1928, 26%) and a minority of deceptive attempts at study enrollment were detected during the screening and enrollment call (18/1928, 1%; see Table 3). Table 4 displays reported characteristics of deceptive attempts at enrollment at the REDCap screener level, screening and enrollment call level, and those that fully enrolled in the study. After all deception checks were complete, 1409 individuals remained eligible to enroll in the study. Ultimately, 924 individuals did not enroll due to various reasons (ie, unable to contact, canceled screening and enrollment call, or ineligible phone) and 485 individuals were randomly assigned into the study. Participants completed 83.8% (28,948/34,552) of all EMAs, and 96.6% (397/411) completed the follow-up assessment. Additional details are provided elsewhere [20].

**Table 3:** REDCap^a^ screener and screener and enrollment call outcomes.

Variables			Value
**Facebook screener clicks, n**			5236
**Incomplete screeners (step 1), n**			1689
**Ineligible due to inclusion criteria not met, n**			1619
**Eligible before deception check, n**			1928
**REDCap deception check, n/N (%)**			501/1928 (26)
**Duplicates (step 2), n/N (%)**			301/501 (60.1)
**Invalid address (step 3), n/N (%)**			156/501 (31.1)
**Invalid phone number (step 4), n/N (%)**			2/501 (0.4)
“**Other” or “No reason” listed, n/N (%)**			42/501 (8.4)
**Screening and enrollment deception check** **, n/N (%)**			18/1928 (1)
	Invalid phone	6/18 (33.3)	
	Duplicates	6/18 (33.3)	
	Invalid address	5/18 (27.8)	
	Evidence of an automated program (bot)	1/18 (5.6)	
**Total eligible to enroll after all deception check, n**			1409
	Fully enrolled and randomly assigned	485	
	Poststudy SSN^b^ check—inaccurate	3	

^a^REDCap: Research Electronic Data Capture.

^b^SSN: social security network.

**Table 4:** Reported demographics by potential deception status. Demographics characteristics listed in the first two columns may not be accurate as deception was detected.

Variable					Flagged as potentially deceptive in prescreener (n=501)		Flagged as potentially deceptive in enrollment call (n=18)		Fully enrolled participants (n=485)
**Age (years), mean (SD)**					43.1 (13.5)		32.4 (6.9)		48.2 (12.4)
**Ethnicity (n, %)**									
		Hispanic		58 (11.6)		2 (11.1)		48 (9.9)	
		Non-Hispanic		443 (88.4)		16 (88.9)		437 (90.1)	
**Race, n (%)**									
		American Indian or Alaska Native		9 (1.8)		0 (0.0)		7 (1.4)	
		Asian		16 (3.2)		0 (0.0)		13 (2.7)	
		Black or African American		191 (38.1)		10 (55.6)		93 (19.2)	
		Native Hawaiian or Other Pacific Islander		1 (0.2)		0 (0.0)		1 (0.2)	
		White		265 (52.9)		7 (38.9)		342 (70.5)	
		More than one race		19 (3.8)		1 (5.6)		29 (6.0)	
**Biological sex, n (%)**									
	Female			289 (57.7)		8 (44.4)		370 (76.3)	
	Male			212 (42.3)		10 (55.6)		115 (23.7)	

## Discussion

### Principal Findings

This study used a systematic deception detection procedure to attempt to preserve study resources, data integrity, and participant safety in a remotely delivered behavioral study. Given the relatively large scale of our digitally mediated recruitment methods, the low risk involved in study participation, considerable financial compensation, and robust practices for ultimately verifying participants’ identity, this study provided unique insight into how deceptive practices may be occurring in large, remotely delivered behavioral studies, as well as how to mitigate their impact. The digitally mediated deception check implemented at the REDCap prescreener level (steps 1-8) resulted in 26% (501/1928) of the eligible sample being excluded. Only 1% (18/1928) of the eligible sample was excluded during the subsequent screening and enrollment call. The systematic deception detection steps detailed in this paper support data integrity and may minimize the risk of deceptive enrollments.

Within the digitally mediated REDCap prescreener steps, duplicate screeners and address checks provided the most benefit to the deception detection process in terms of the number of potentially deceptive instances identified. Study staff sorted and compared the data and metadata associated with various submissions. The practices employed at this step were similar to those that have been used elsewhere [7]. Some of the submissions removed during the REDCap prescreener level appeared to have been initiated by automated programs (ie, “bots”). This led to the decision to implement a reCAPTCHA checkpoint before allowing potentially eligible individuals to proceed with the prescreener survey. This practice has been successfully used in other remotely conducted research studies [17,18].

In contrast to other studies, we did not exclude individuals on the basis of their IP address. The university institutional review board did not allow us to save and track IP addresses. Further, while IP addresses can provide information useful for determining if an individual is submitting multiple prescreeners from the same device, location-based decision rules may limit enrollments unnecessarily. For example, many companies, academic institutions, and individuals use roaming IP addresses. This can entail a limited number of IP addresses being associated with one large organization or IP addresses that regularly change for a given user. The use of roaming IP addresses can increase security and preserve privacy. Thus, while IP address information may be an important part of identifying potentially deceptive practices, caution should be used to avoid unnecessarily prohibitive practices.

While individuals were not excluded from this study if they had a bandwidth or voice over internet provider (VoIP) phone number, we observed the limited abilities of such devices to interact with the study app. Bandwidth and VoIP phone numbers can be used to mask true phone numbers or to create an internet-based phone number without a physical phone. Bandwidth and VoIP phone numbers are generally not considered “smartphones” and are typically not able to download phone apps. In this study, people with bandwidth phone numbers were allowed to proceed to the enrollment phone call, but we have since decided to exclude them from our studies due to incompatibility. Researchers should carefully consider how they will handle problematic smartphone types and phone number-masking services in mHealth studies, as they are increasingly commonplace and have a bearing on app compatibility and how surveys and notifications are received.

Few attempts at deceptive enrollment were detected at the screening and enrollment call level. This may provide evidence in support of the steps we took to prevent deceptive enrollments at the REDCap prescreener level. During the REDCap prescreener, individuals were informed that identification would be verified before enrollment; the mode of verification, however, was not disclosed. Requiring photo identification and ensuring addresses entered into the prescreener assessment matched the photo identification allowed for a relatively quick and easy way to verify an individual’s identity. To remain compliant with institutional policy, individuals were also required to provide their SSN during the screening and enrollment call. While SSNs could not be checked in real time, we ultimately observed that of the 202 of participants who provided this information and had their SSNs checked, 98.5% (n=199) provided accurate information. Systematically checking SSNs before full study enrollment would likely reduce deceptive enrollments into research studies but would come with costs (eg, staff time and effort, some qualified individuals may refuse to share their SSN).

Some of the deception detection methods that were used in this study were previously employed in other studies [7]. However, some deception detection strategies were novel to this study (eg, requiring photo identification in a remotely conducted trial and conducting phone number checks). Study results demonstrated that using multiple deception abatement strategies can mitigate deceptive enrollments in remotely conducted clinical trials. Future studies should implement similar strategies and further innovate to reduce deceptive attempts at enrollment into clinical trials.

### Limitations

The true sensitivity and specificity of our deception detection and abatement processes are unknown. It is possible that some individuals may have successfully used deception to enroll in the study, and it is possible that some individuals were unnecessarily excluded. In order to deceptively enroll in the study, individuals would have had to fake their identification, mailing address, phone number, and SSN. Following recommended practices to preserve data integrity [19], we conservatively disqualified individuals who had been flagged as potentially engaging in deceptive practices. It is possible, however, that some qualified individuals were excluded from participating in the study.

### Future Directions

One of the goals of this work is to inform the development of more sophisticated, digital solutions to deception detection and abatement. Using automation rather than manual deception detection may increase accuracy and reduce staff burden. Further, sensors (eg, GPS) could be integrated into deception detection procedures to verify home addresses and participant locations. Future studies should calculate study costs and potential cost savings of implementing deception detection and abatement procedures.

### Conclusions

We implemented a systematic deception detection procedure to reduce the likelihood of deceptive enrollments into a nationwide RCT. To maintain data integrity and participant safety, we recommend engaging in robust identification practices with study participants in a live enrollment call. Before this phase, personnel and financial resources can be preserved by implementing systematic, digitally mediated processes to flag cases of potential deception. Technologies and study operating procedures centered on analyzing incoming screening survey data and their associated metadata can help to efficiently screen out instances of likely deception. More research is needed to improve upon and automate these processes, and researchers must remain vigilant to the advent of new threats. While deception detection procedures are primarily aimed at reducing the likelihood of enrolling individuals who do not actually meet the study inclusion criteria, they can also positively impact staff time, motivation, job satisfaction, and the study budget.

## Abbreviations

CAPTCHA: Completely Automated Public Turing test to tell Computers and Humans Apart
EMA: ecological momentary assessment
mHealth: mobile health
OUHSC: University of Oklahoma Health Sciences Center
RCT: randomized controlled trial
REDCap: Research Electronic Data Capture
SSN: social security number
VoIP: voice over internet protocol

## Data Availability

The datasets generated during and/or analyzed during this study are available from the corresponding author upon reasonable request.
